# Contribution of Nanoscience Research in Antioxidants Delivery Used in Nutricosmetic Sector

**DOI:** 10.3390/antiox11030563

**Published:** 2022-03-16

**Authors:** Irene Dini

**Affiliations:** Pharmacy Department, “Federico II” University, Via D. Montesano, 49, 80131 Naples, Italy; irdini@unina.it

**Keywords:** nanotechnology, nutraceutic, nutricosmetic, nanoceutic, phytochemical delivery, nanoemulsion, polymeric nanoparticles, edible nanocoating, nanocosmeceuticals

## Abstract

Nanoscience applications in the food and cosmetic industry offer many potential benefits for consumers and society. Nanotechnologies permit the manipulation of matter at the nanoscale level, resulting in new properties and characteristics useful in food and cosmetic production, processing, packaging, and storage. Nanotechnology protects sensitive bioactive compounds, improves their bioavailability and water solubility, guarantees their release at a site of action, avoids contact with other constituents, and masks unpleasant taste. Biopolymeric nanoparticles, nanofibers, nanoemulsions, nanocapsules, and colloids are delivery systems used to produce food supplements and cosmetics. There are no barriers to nanoscience applications in food supplements and cosmetic industries, although the toxicity of nano-sized delivery systems is not clear. The physicochemical and toxicological characterization of nanoscale delivery systems used by the nutricosmeceutic industry is reviewed in this work.

## 1. Introduction

Nutricosmetics is a new sector of cosmetics that aims to optimize the use of cosmetic products and food supplements such as micronutrients (minerals, vitamins), macronutrients (peptides, essential fatty acids), and botanicals (herbal and fruit extracts) to nourish the skin and reduce skin aging through an integrated “In and Out” approach [1,2,3,4,5]. By 2030, the world’s population over 60 years will grow to 1.4 billion [6], so it is reasonable to assume that the number of people who will buy cosmetics in hopes of maintaining a youthful appearance will grow in the coming years. In this scenario, nutricosmetic products should find a large market as they are natural products, improve health, and are considered free of side effects. The skin is the first line of defense between our body and the world [7]. It maintains the balance of liquids by binding water, preventing its loss, and promoting perspiration. The skin is subjected to multiple stressors that lead to premature skin aging. Free radicals produced by air pollution, cold, and UV rays induce inflammatory processes and accelerate skin aging by altering our body’s DNA, lipids, and proteins [8]. Sportswear can produce dryness and irritable skin, increasing friction. Frequent showers and detergents modify the hydrolipidic film and the ability to regulate liquids and the skin’s elasticity. The nutricosmetic approach repairs the skin barrier [9], improves skin hydration, fights inflammation, and protects the skin from damage caused by the sun’s rays, combining food supplements that intervene from the inside with cosmetic products for topical use, which interfere from the outside [10]. Nanotechnological systems are enjoying great success in the food and cosmetic fields. In recent years, new delivery systems for bioactive compounds have been investigated. The purpose of this work is to review the physicochemical and toxicological impact of nanoscale delivery systems used by the nutricosmeceutic industry.

## 2. Nanocosmetics and Nanonutraceuticals Delivery Systems

Nanochemicals are formulations containing nanotechnology as delivery systems to improve the bioactive components’ performance [11,12,13]. REACH (Registration, Evaluation, Authorisation, and Restriction of Chemicals) regulates the exposure and hazards of nanochemicals [14].

Nanocosmetics is the cosmetic field where nanomaterials/nanoparticles are used to develop cosmetic products. The international guidelines (EC Regulation 1223/2009) that guarantee the protection and safety of cosmetic products defined nanomaterials as only the “material insoluble or bio-persistent (e.g., metal oxides, metals, etc.) and intentionally manufactured with one or more external dimensions, or an internal structure, on the scale from 1 to 100 nm”, excluding materials that are soluble, degradable, and/or non-persistent in biological systems (e.g., liposomes, plant-derived vesicles, emulsions, etc.) [15].

Nanonutraceuticals are nanotechnology delivery systems used to improve the performance of bioactive components in foods, including food supplements. An edible delivery system must be realized with GRAS (Generally Recognized as Safe) ingredients by using processing operations that conform to good manufacturing practices. It must have a high loading capacity, encapsulation efficiency, and retention efficiency. It must have the capacity to protect chemically labile encapsulated compounds from chemical degradation (e.g., oxidative degradation) [16]. It must be compatible with the food or beverage matrix that it will be incorporated into, without causing any adverse effects on product appearance, texture, mouthfeel, flavor, or shelf-life [17]. It must be resistant to environmental stresses during production, storage, transport, and utilization (e.g., thermal processing, light exposure, mechanical agitation, chilling, freezing, or dehydration) [17]. It must be designed to control the release and/or absorption of the bioactive lipophilic component of a particular site within the gastrointestinal tract, such as the mouth, stomach, small intestine, or large intestine [17].

In the nutricosmetic field, nanotechnology is used to prepare sunscreens, barrier creams, antiacne, moisturizers, antiaging, antioxidants, hair, nails, lip, and skin cosmetics. The industry has created many nanoscale delivery systems that transport each bioactive based on its nature (lipophilic and hydrophilic) and chemical–physical properties. Nanostructures may have one-dimension, two-dimensions (e.g., nanotubes), or three dimensions (nanoparticles) at the nanometer level [18]. Nanostructures that protect and deliver lipophilic compounds include simple oil in water (O/W) emulsions, water-in-oil-in-water (W/O/W) double emulsions, capsules, liposomes, and colloidosomes. The systems able to protect and deliver hydrophilic bioactive components are gelled networks (hydrogels), gel particles/fluid gels (gelled nanoparticulates), water in oil emulsions (W/O), and protein–polysaccharide structures (self-assembled structures).

### 2.1. Nanodispersions

#### 2.1.1. Nanoemulsions

Nanoemulsions (also known as miniemulsions or submicron emulsions) are metastable colloidal dispersions with average droplet radii ranging from 10 to 100 nm. They can be made to be highly viscous or gel-like. They are optically transparent, since their small dimensions disperse light waves imperceptibly [19]. Two types of nanoemulsions exist: O/W (oil/water) able to encapsulate, protect, and deliver hydrophobic functional components such as lipophilic vitamins (e.g., β-carotene), omega-3, and nutraceutical (e.g., plant sterols, carotenoids, etc.) essential oils; and W/O (water/oil) used to encapsulate water-soluble active agents such as polyphenols, emulsifiers (phospholipids, Tweens, proteins), texture modifiers (pectin, sodium alginate, carrageenan), and preservatives (parabens) [20]. One or more emulsifiers is used to decrease the energy required to form smaller droplets, reduce the interfacial tension, and prevent or slow down the aggregation of particles of the dispersed phase by increasing repulsion forces between them [21]. Examples of food-grade emulsifiers are proteins and polysaccharides [22]. The silicone oil-in-water nanoemulsions are used to enhance the silicone oil deposition on the hair surface to preserve the moisture and lubrication of hair [23]. The droplet size depends on the approach, the operating conditions (e.g., energy intensity, time, and temperature), and the system’s composition (e.g., interfacial tension and viscosity). Nanoemulsions may be formed using either high or low energy (phase inversion and spontaneous phase separation) techniques. The high-energy approaches employ homogenizers, microfluidizers, and sonicators [24]. The low energy methods (physicochemical approaches) are PIT (phase inversion temperature), PIC (phase inversion composition), and spontaneous emulsification.

In the cosmetic field, nanoemulsions are used to increase the delivery of active compounds in the skin, avoid cream in fluid products, and maintain the transparency and gloss after spreading [25]. Examples are O/W nanoemulsions containing *Opuntia ficus indica* (L.) extract used as moisturizing agents [26], nanoemulsions containing hydroalcoholic extracts of *Vellozia squamata* leaves employed as antiaging agents [27], and pomegranate seed oil nanoemulsions prepared to enhance antioxidant activities in the skin and to protect against photodamage [28].

Resveratrol is encapsulated in nanoemulsions in food supplements to protect it from UV exposure [29].

(1) The high-energy methods used to produce nanoemulsions

The high-energy methods are high-pressure homogenization, microfluidification, and sonication.

High-pressure homogenization (HPH) is a purely mechanical process induced by forcing a fluidic product through the homogenizing nozzle at high pressure (150–200 MPa, or 350–400 MPa for ultra-high-pressure homogenization (UHPH)) [30]. HPH is used to make O/W liquid nanoemulsion in which the oil phase is less than 20%. It can decrease the polydispersity of oil droplets and the droplet size [31]. It is inappropriate to formulate creamy or high viscosity nanoemulsions whose droplet diameters are below 200 nm [32]. Tiny oil droplets of an extract of jackfruit, obtained by HPH at 800 bar, were used to formulate a cream with low viscosity and high stability [33].

The microfluidizer works on the principle of pressurized stream. It uses a pump to force a coarse emulsion pre-mix through a narrow orifice at high pressures to facilitate droplet disruption. The channel is designed to split the coarse emulsion into two streams made to impinge on each other at high velocity in an interaction chamber [34]. Smaller emulsions can be made by enhancing the pressure up to ~700 MPa [35]. It produces smaller and narrower particle sizes of nanoemulsions than the HPH [36].

Sonication is a process in which sound waves (high-intensity ultrasonic wave frequency >20 kHz) are used to agitate particles in solution. Such disruptions can be used to form emulsions containing very fine droplets, mix solutions, and speed the dissolution of a solid into a liquid. A nanoemulsion containing avocado oil droplets obtained by the ultrasonication technique was used by Silva et al. (2013) to formulate a sun protector [37].

(2) Low-energy methods used to produce nanoemulsions

Low energy methods are based on the spontaneous dissolution of hydrophobic substances (oil, lipophilic surfactant, and water-miscible solvent) in an organic solvent, which is further emulsified with an aqueous solution (hydrophilic surfactant and water) [38].

In the PIT method, the nanoemulsions are spontaneously made by changing the temperature profile of the components. Low interfacial tensions (102–105 mNm^−1^) are used to promote the emulsification process [39].

In the PIC method, a phase inversion occurs when the continuous phase is mixed over the component that will make the dispersed phase, and the chemical energy from the reaction of the components forms a fine dispersion [40].

The spontaneous emulsification is obtained, putting a lipid phase containing the surfactant in the aqueous phase under nonstop magnetic stirring and removing the aqueous phase under reduced pressure [41].

Low-energy methods were used to make a micellar formulation based on an extract of *Vellozia squamata* with antioxidant properties [27].

#### 2.1.2. Nano-Double Emulsions

The double emulsions are compartmentalized liquid dispersions in which the droplets of the dispersed phase contain smaller droplets of similar composition (but not necessarily identical) as the continuous phase. The double emulsion structures can be water-in-oil-in-water (W/O/W) or oil-in-water-in-oil (O/W/O). Concerning the W/O/W type, the three distinct phases consist of internal water droplets dispersed in an oil phase, then enclosed in a continuous water phase. An emulsifier with low interfacial tension stabilizes the interface between two immiscible liquids. The W/O/W emulsions can carry both polar and nonpolar bioactive compounds. They are used as a delivery system of flavors, phytochemicals, probiotics, hydrophilic (i.e., water-soluble vitamins, minerals), and hydrophobic (i.e., polyunsaturated fatty acids) nutrients [42,43]. Finally, the multiple emulsions O/W/O and W/O/W are used to separate two aqueous phase components that might adversely react with each other if they were present in the same aqueous phase and to protect and to release an aqueous phase component locked in the inner phase to an exact site, such as the mouth, stomach, or small intestine. Recently, an anti-pollution cosmetic containing D-biotin based on a W/O/W multiple emulsion was formulated for skin protection by Ali et al. [44].

(1) Methods used to produce W/O/W nanoemulsions
  - Bulk emulsification methods
    W/O/W nanoemulsions are produced using a two-step emulsifying process. The first step creates the smallest possible internal droplets (water-in-oil emulsion) by high shear conditions. The second step is carried out under lower energetic conditions to prevent rupture of the primary emulsion. It consists in dispersing and emulsifying the mixture in water, using a combination of surfactants and shear to create a stable, aqueous emulsion. Two types of surfactants are needed: a low hydrophilic–lipophilic balance (HLB) for the interface between the interior water droplet and the encompassing oil droplet; and a high HLB for the interface between the oil droplet and continuous water phase [45]. The multiple emulsions are thermodynamic unstable. Biopolymers are used to minimize the leakage of the encapsulates from the internal aqueous phase, flocculation of the droplets, or phase separation during processing and storage of the Pickering particles. Biopolymers such as gelatin, caseinate, whey protein, bean protein, gum acacia, xanthan gum, and gelled starch stabilize the droplets in the internal phase of W/O/W food emulsions. Instead, polysaccharides such as carrageenan, locust bean gum, xanthan gum, pectin, gum arabic, whey protein isolate, sodium caseinate, egg white powder, and microcrystalline cellulose are utilized to stabilize the droplets of secondary emulsions [46]. Other stabilizers used are texture modifier sugars (e.g., sucrose, HFCS), polyols (e.g., glycerol, sorbitol), polysaccharides (e.g., xanthan, pectin, carrageenan, alginate), and proteins (e.g., whey protein isolate, gelatin); weighting agents such as dense lipophilic materials (e.g., brominated vegetable oil, sucrose acetate isobutyrate, ester gums); and ripening retarder such as lipophilic materials with very low water-solubility (e.g., ester gums) [47,48]. The W/O/W double Pickering emulsions containing silica particles and carboxymethyl cellulose as stabilizers were used in polymer particles with closed-cell porous and uniform size [49].
    A phase-inversion method was optimized to create a multicore W/O/W nanoemulsion for dermal delivery of acyclovir [49].
  - Microfluidic techniques
    The microfluidic device’s technology is proposed to produce a double emulsion as an alternate and versatile route. Capillary microfluidic devices consist of coaxial assemblies of glass capillaries on glass slides. One of the advantages of these devices is that their irritability can be easily and precisely controlled by a surface reaction with an appropriate surface modifier. In contrast to bulk emulsification methods, the emulsion in a microfluidic device is made by precisely fabricating one drop at a time. This process results in a highly monodispersed emulsion. One of the most attractive features of microfluidic techniques is that they enable the fabrication of double, triple, and even higher-order emulsions. The size and number of the encapsulated droplets can be manipulated (droplet sizes from 2 µm to 250 µm in diameter). Hydrophilic and hydrophobic surfaces, as well as surfactants, stabilize the two-fluid interfaces, extending manufactured products’ stability. The flowing conditions can control the dispersity and size of droplets. The productivity is limited [50]. Lee et al. prepared a W/O/W double Pickering emulsion by a capillary microfluidic device in which the ellipsoidal double emulsion droplet formed a peanut-like colloidosome, enhancing the number of inner droplets [51].

#### 2.1.3. Association Colloids

The association colloids such as surfactant micelles, vesicles, bilayers, reverse micelles, and liquid crystals are stable micro heterogeneous systems (dimension range of 5 to 100 nm) in which the particles of the dispersed colloidal phase are typically transparent solutions. They are formed by molecules or ions dissolved in the dispersion medium containing small polar, nonpolar, and/or amphiphilic functional particles.

The micelles are relatively simple spherical or rod-like structures that consist of a phospholipid double layer. The encapsulation in O/W micelles allows for the inclusion of lipophilic compounds into aqueous systems. The encapsulation of compounds in W/O nanoemulsions or reversed micelles introduces hydrophilic compounds into a lipophilic environment. Their formation is spontaneous (thermodynamically favorable), and it is determined by the hydrophobic effect obtained by reducing the contact area between the nonpolar groups of the surfactant and water. The temperature, ionic strength, pH, and concentration and molecular characteristics of the surfactant and cosurfactant affect the type of colloid formed. Colloidal delivery systems can be classified into self-assembled molecules, solid-in-liquid, and liquid-in-liquid dispersions. The bioactive compounds can be incorporated as a solution (soluble form) or dispersion (insoluble form). The chemical reactivity of the soluble component increases its absorption, bio-accessibility, and taste characteristics [52]. Recent advances showed the utility of using colloids (lipid-origin carriers and nanoemulsion) as photosensitive nanocarriers in cosmetic formulations [53].

### 2.2. Nanoparticles

Nanoparticles are colloidal-sized particles composed of polymers with diameters ranging from 10 to 1000 nm. They are grouped into solid lipid nanoparticles, polymeric nanoparticles, ceramic nanoparticles, hydrogel nanoparticles, copolymerized peptide nanoparticles, nanocrystals and nanosuspensions, nanotubes and nanowires, functionalized nanocarriers, nanospheres, and nanocapsules [54].

The nanoparticle delivery systems improve skin penetration, release, and surface functionalization of active compounds. They may contain metallic ions. Metallic nanoparticles, which contain iron oxide, gold, silver, gadolinium, and nickel, are used to formulate cosmetics, sunscreens, and personal care products [54]. For example, sunscreens containing ZnO or TiO_2_ nanoparticles are more transparent than micron-sized formulations [55]. It was shown that the safranal-nanoparticles (solid–lipid nanoparticles) improve sun screening activity in the range of 103–230 nm [56]. Nail paints having nanoparticles enhance mar resistance, toughness, and impact resistance of the mammalian nails [57].

In nutraceutical preparations (including food supplements), nanoparticles are used to improve the bioaccessibility of resveratrol [58] and folic acid [59] and the antioxidant properties of bioactive compounds [60].

#### Methods Used to Produce Nanoparticles

(1) Desolvation

Desolvation is a thermodynamically driven, self-assembly process for polymeric materials. The addition of desolvating agents (i.e., salts, ethanol, acetone) separates and coacervates the polymeric molecules in the aqueous phase. The self-assembly of the polymer molecules occurs with electrostatic interactions, since the overall free energy in the system is minimized during desolvation. The polymeric molecules form particles of different shapes and sizes depending on the preparation conditions. A balance between attractive and repulsive forces is necessary for fabricating particles of an appropriate size. The suppression and expression of hydrophobic interactions provide a way to control the size of polymeric particles during desolvation [61].

Duclairoir et al. made nanoparticles based on gliadins with vitamin E to improve the interaction between vitamin E and epidermal keratin [62].

(2) Electrospray drying technique

A syringe pump slowly injects a conducting liquid through a needle on which an electrical potential is applied. The electric stress accelerates the liquid away from the needle. The nanoparticles are produced when the solvent is evaporated [63].

Zaeim et al. used the electrospray technique to encapsulate probiotics and showed that the acacia gum concentration affects the viscosity, and the concentration of Tween-80 affects the form of the particles [64].

### 2.3. Nanocapsules

Nanoencapsulation is a technology used to package flavoring agents (e.g., sweeteners, seasonings, spices, essential oils), food acids and bases (e.g., citric acid, sodium bicarbonate), lipids (e.g., vegetable oils, milk fat), food additives (e.g., preservatives, pigments), minerals (e.g., calcium and iron salts), vitamins (e.g., carotene), colors, omega-3 oils, phytochemicals, and probiotic bacteria. The industry produces single-core and multiple-core nanocapsules. Single-core nanocapsules have high core loading (e.g., 90% of the total capsule weight). They are obtained by complex coacervation, fluidized bed drying, droplet coextrusion, and molecular inclusion. The storage stability is obtained using high-pressure homogenization and/or a surfactant.

In cosmetics, nanocapsules are employed in antiaging and moisturizing creams [65]. They can be applied to the skin or incorporated in semisolid formulations. After topical application on the skin, nanocapsules create a thin film with water evanescence responsible for the long-term delivery and increased storage capacity of the bioactive compound [65]. The most used biodegradable polymers in skincare formulations are aliphatic polyesters (i.e., poly(ε-caprolactone) (PCL) and poly(lactic acid) (PLA)), the chitin a polysaccharide found in the exoskeleton of crustaceans and insects, the gelatin which consists of a mixture of high molecular weight proteins, and the hyaluronic acid [66]. The multiple core nanocapsules are principally produced by spray drying, the core material is dispersed throughout the wall material, and the central area is occupied by the void resulting from the expansion of particles during the later drying stages.

Examples of nano-capsular vehicles for nutraceuticals are casein micelles used to deliver calcium phosphate and protein, stabilize hydrophobic substances, and enrich non-fat or low-fat food products; and poly(lactide-co-glycolide) micelles employed to carrier essential oils [67].

#### Nanoencapsulation Techniques

The nanoencapsulation techniques can be used to encapsulate hydrophilic and lipophilic bioactive compounds. Top-down and bottom-up approaches are employed to make nanoparticles.

The top-down approach is a mechanical process that uses shear or particle collisions as the energy source to break down larger entities into smaller aggregates [68].

The bottom-up (or self-assembly method) uses chemical or physical forces operating at the nanoscale to assemble lipids and proteins into larger structures. The pH, temperature, concentration, and ionic strength affect the process [68]. The emulsification, coacervation, and supercritical fluid techniques are used to encapsulate the hydrophilic and lipophilic compounds. The inclusion complexation, emulsification–solvent evaporation, and nanoprecipitation techniques are mainly used for lipophilic compounds [68].

Both top-down and bottom-up approaches have been utilized to deliver carotenoids in food and cosmetic formulations [69].

(1) Emulsification Technique

Emulsion technology is applied to encapsulate bioactive compounds in aqueous solutions. Nanoemulsions are made using high-energy emulsification methods. They possess high kinetic stability due to tiny emulsion droplet sizes. Nanoemulsions can either be used directly in the liquid state or be dried to powder form using drying techniques such as spray drying and freeze-drying after emulsification [70].

(2) Emulsification–solvent evaporation

In the solvent evaporation process, the polymer is dissolved in a suitable water-immiscible solvent, and the supplement or the nutrient is dispersed or dissolved in this polymeric solution. The organic solvent diffuses into the aqueous phase and evaporates at the water/air interface. The solution or dispersion is emulsified in a continuous aqueous phase to form the droplets. The nanospheres can be obtained after filtration and drying [70].

(3) Supercritical fluid technique

Supercritical fluids are used for the encapsulation of thermally sensitive compounds. This technique employs low critical temperature and low organic solvent levels. The bioactive compound and the polymer are solubilized in a supercritical fluid. The solution is expanded through a nozzle, the supercritical fluid is evaporated in the spraying process, and solute particles are precipitated. The most common processing techniques involving supercritical fluids are supercritical antisolvent (SAS) and rapid expansion of critical solution (RESS). The process of SAS uses a liquid solvent to dissolve the solute (e.g., methanol) utterly miscible with the supercritical fluid and a supercritical fluid. The CO_2_ is the most widely used supercritical fluid because of its mild critical conditions (Tc = 31.1 °C, Pc = 73.8 bars), nontoxicity, nonflammability, and low price. The extract of the liquid solvent by supercritical fluid leads to the instantaneous precipitation of the solute (the solute is insoluble in the supercritical fluid), resulting in the formation of nanoparticles. The RESS differs from the SAS process because its solute is dissolved in a supercritical fluid (such as supercritical methanol), and the solution rapidly expands through a small nozzle into a region at lower pressure. In this procedure, the precipitate is solvent-free [71]. Dai et al. used nanoparticles containing curcumin in zein–lecithin composite obtained by antisolvent coprecipitation to improve the stability of curcumin against UV, thermal treatment, and high ionic strength [72].

(4) Complex coacervation

Complex coacervation is a spontaneous phase separation process involving two liquid phases (acetone or ethanol) in colloidal systems, which results from the interaction of two oppositely charged polyelectrolytes upon mixing in an aqueous protein solution. The formation of coacervates depends on the substrates, pH, temperature, molecular weight, ionic strength, and polyelectrolyte concentration and are limited by the addition of a crosslinking agent, such as glutaraldehyde. There are simple and complex coacervation methods. In simple coacervation, a solute phase is moved to the coacervation phase by changing the temperature, ionic strength, molecular weight, pH, and electrostatic interaction. Complex coacervation is obtained by mixing two ions of opposite charge into two immiscible liquid phases. The electrostatic interactions control the structure during the synthesis. The coacervation methods use a cationic (e.g., calcium chloride) or polyanionic (e.g., sodium tripolyphosphate) counter-ion, and a biodegradable hydrophilic polymer (e.g., chitosan, sodium alginate, and gelatin) [73] with a positive or negative charge in which the bioactive component is included. A polymer is added that allows polyelectrolyte complexation and nanocapsule formation [74].

Polysaccharide–protein complexes are used as carriers for encapsulating active compounds in supplement and cosmetic formulations. Coacervation is used to encapsulate polyphenols and essential oils [75].

(5) Inclusion complexation

Inclusion complexation encapsulates a supra-molecular association of a ligand (encapsulated ingredient) into a cavity-bearing substrate (shell material) through hydrogen bonding, van der Waals forces, or an entropy-driven hydrophobic effect. Inclusion complexation is used for lipophilic molecules (i.e., essential oils and vitamins) [76]. Only a few particular molecular compounds, such as β-cyclodextrin and β-lactogloglobulin, are suitable for encapsulation through this method [77].

Scalia et al. used this technique to produce a sunscreen entrapping 2-ethyl-hexyl-p-dimethylaminobenzoate-DMAB into the hydroxypropyl-β-cyclodextrin cavity to improve its photostability [78].

(6) Nanoprecipitation

The nanoprecipitation method is also called solvent displacement. This method is used to encapsulate lipophilic compounds. It yields stable aqueous suspensions of nanoparticles of about 300–320 nm based on the spontaneous emulsification of water [79].

Jummes et al. formulated poly-ε-caprolactone nanoparticles loaded with *Cymbopogon martinii* essential oil to use as antioxidants in the cosmetic field [80].

### 2.4. Nanohydrogels

Hydrogels are hydrated polymer gels formed by three-dimensional macromolecular networks that swell but do not dissolve in water. They are obtained from natural polysaccharides such as dextran, pullulan, or cholesterol-containing polysaccharide. Nanohydrogels have tiny dimensions, usually 20–30 nm. The reduced size of nanohydrogels enables the dispersion of water-insoluble additives (e.g., flavors, colors, and preservatives) [81]. Nanohydrogels can be produced either by physical or chemical gelation. The physical hydrogels (also called “reversible” or “pseudo” gels, physical nanohydrogels) exhibit high water sensitivity (degrade and even disintegrate entirely in water) and thermo-reversibility (melt to a polymer solution when exposed to heat) [82]. The chemical nanohydrogels (also called “irreversible” or “permanent” gels) are networks of polymer chains covalently linked at strategic connection sites. The chemical nanohydrogels neither disintegrate nor dissolve in an aqueous solution and have viscoelastic properties. They act as foaming and emulsifying agents.

Protein–polysaccharide nanohydrogels are used to encapsulate hydrophobic nutraceuticals [82]. A protein-based nanohydrogel was proposed by Bourbon et al. to encapsulate lipophilic compounds such as curcumin and hydrophilic compounds such as caffeine [83].

### 2.5. Solid Lipid Nanocarriers (SLNs) and Lipid Nanocarriers (NLCs)

SLNs are prepared from solid lipids (e.g., glyceryl behenate, stearic triglyceride, acetyl palmitate, and glycerol tripalmitate), while NLCs are composed of solid and a tiny amount of liquid lipids. Generally, biomolecules are incorporated between the fatty acid chains, lipid layers, or amorphous clusters. Both the SLNs and NLCs are stabilized by surfactants and can improve the bioavailability of highly lipophilic molecules. They are valuable in preparing the topical and transdermal application of poorly water-soluble and/or unstable compounds [84]. SLNs have a skin hydration effect and enhance the penetration through the stratum corneum since the surfactant(s) act as a permeation enhancer. NLCs have higher loading capacity and physicochemical stability during storage than SLNs [85,86]. Neem oil–solid lipid nanoparticles are formulated to be antibacterial on acne microbes [87]. Tretinoin- and behenate solid lipid nanoparticle-based gels have been made to reduce skin irritation and improve occlusive effects in cosmetic preparations for treating acne [88]. Solid lipid nanoparticles and nanostructured lipid carriers containing ascorbyl palmitate are utilized to improve moisturizing effects [89].

Lipid nano-delivery systems containing genistein, resveratrol, curcumin, quercetin, and epigallocatechin-3-gallate modulate chronic inflammation, oxidative stress, and aging-associated disorders [90]. Nano-lipoidal carriers of isotretinoin are used in cosmetics to combat aging [91].

Many techniques are used to make SLNs and NLCs. In some instances, different methods are employed together to prepare the nanoparticles. The high-pressure homogenization (HPH) and microemulsion techniques are the most used [84].

### 2.6. Nanoliposomes

Liposomes are spherical colloidal structures (0.05–5 μm) created with one or more lipid bilayers, (phospholipids, such as lecithin) by supplying energy (by sonication, homogenization, heating, etc.), which contain an aqueous solution in the core. Stabilizing agents, such as sterols (e.g., cholesterol), are used to increase liposomes’ stability. A significant advantage of nanoliposomes is that they can simultaneously incorporate and release two materials with different solubilities (bifunctional liposomes). They can be utilized in the entrapment, delivery, and release of water-soluble and lipid-soluble functional components such as peptides, enzymes, vitamins, and flavors. Nanoliposomes can be made by supercritical fluid (CO_2_), high-pressure homogenization, and micro fluidization techniques [92].

In cosmetic products, nanoliposomes are used to formulate antiaging creams, sun lotions, moisturizers, lipsticks, treatment of hair loss, and facial beauty masks [12]. Tan et al. showed that lutein, β-carotene, lycopene, and canthaxanthin encapsulation into liposomes enhanced their antioxidant activity [93]. The lipsticks containing rice bran oil liposomes showed better stability and antioxidant properties than conventional ones [94].

In nutraceutical preparations, nanoliposomes improve curcumin stability at the alkaline condition and temperature variation [95] and the antioxidant activity of phenolic compounds extracted from olive leaves [96].

### 2.7. Nanoclay

“Nanoclay” is obtained by clay minerals or layered silicates with metal oxides and organic matter traces. The clay minerals are hydrous aluminum phyllosilicates with variable magnesium, iron, alkaline earth, alkali metals, and other cations. Synthetic and natural clays are commercially available. Natural clays are made with “SiO_2_” and “AlO_6_” units [97]. For example, kaolite is made with “SiO_2_” and “AlO_6_” units in ratios of 1:1, montmorillonite and vermiculite in ratios of 2:1, and chlorite in a ratio of 2:2 [97]. Clay minerals are used in wound healing formulations [98]. Mg-rich smectite clay mineral helps in collagen synthesis and angiogenesis on skin wounds [99]. Palygorksite and sepiolite showed anti-inflammatory properties [100]. Clay minerals are used to formulate thermal muds (nanoclay/spring water hydrogel) [101] employed in skincare products for degreasing, cleansing, exfoliating, invigorating, hydrating, and firming activities [102].

## 3. The Use of Antioxidants in Nutricosmetic Products

Nutricosmetics is an umbrella term for food supplements with aesthetic benefits beyond their primary nutritional value. They are considered nonpharmaceutical and nonmedicinal products, although they are sold in capsules, tablets, syrups, gels, solutions, and extracts. Nutricosmetic supplements can contain nutrients and secondary plant metabolites (also known as phytochemicals or botanicals) [103,104]. Phytochemicals are non-nutritive plant chemicals with protective or disease preventive properties such as antioxidant activity, antimicrobial effects, hormone metabolism modulation, immune system stimulation, and anti-aggregate action. They are considered non-essential nutrients since the human body does not require them for sustaining life. The great changeability of phytochemical compounds determines a significant variation in their physicochemical properties (e.g., solubility in water or oil medium) [105]. The health effects depend on absorption, distribution, metabolism, and excretion. The absorption depends on the dose, the matrix in which they are ingested, and the presence of compounds able to bind or solubilize phytochemicals, reducing their bioactivity or product stability. Some phytochemicals are present in plant foods, such as glycosides or other conjugates, and must be hydrolyzed to be absorbed. Their metabolism may be affected by environmental exposures, stability, activity, gut microbials, and variations in levels of endogenous compounds that modulate biotransformation pathways [106,107]. In particular, among phytochemicals, antioxidant compounds such as vitamins (i.e., E, A, and C), tocopherols, carotenoids, methylxanthines (theophylline, caffeine, and theobromine), and phenols have been shown to improve our aesthetic wellbeing, making anti-inflammatory, antioxidant, photoprotective, antiaging, antiviral, and antibacterial effects [10,108,109]. The combination of topical application cosmetics and oral intake products enhances the results [110]. Both synthetic and natural molecules are employed in nutricosmetic products [111]. Butylated hydroxytoluene (BHT), butylated hydroxyl anisole (BHA), and propyl gallate are examples of synthetic antioxidants. Some synthetic antioxidants are obtained from natural ones. Polyphenols, mineral antioxidants (i.e., selenium, iron, copper, manganese, and zinc), vitamins, and phyto-antioxidants are natural compounds used in cosmetic products. Synthetic and natural antioxidants can be used together to produce synergistic stabilization effects [112]. Antioxidants can be grouped into non-enzymatic and enzymatic compounds [113]. Generally, their levels depend on the types of skin cells. For example, melanocytes do not contain enzymatic antioxidants [114]. The biopharmaceutical classification of antioxidants is based on their permeability and solubility. Four classes of antioxidants are estimated: high solubility–high permeability (i.e., vitamin C, are located in cellular fluids); low solubility–low permeability; low solubility–high permeability; and high solubility–low permeability (i.e., vitamin E, are present in cell membranes) [115]. The administration of antioxidant compounds involves overcoming different obstacles depending on whether they are administered for oral or topical use. The biological activity of the antioxidants administered orally is negatively influenced by the low solubility in the gastrointestinal fluids and aqueous media, instability at physiological pH, and degradation due to enzymes and light. Efficient delivery systems which can enhance their bioavailability are micelles, nanoemulsions, nanoparticles, nanocochleates, nanocapsules, nanocrystals, etc.

In cases where they are used in preparations for topical use, the main problems are instability, low permeability, and water-solubility. The instability is due to environmental stress (i.e., air, light, moisture, heat, oxygen, metal ions, and alkalinity) and determines the shelf life of the products [112]. The low permeability and water-solubility negatively affect their ability to enter into more profound layers of the skin and arrive at the target tissue [116]. For example, the use of resveratrol is limited in cosmetic formulations due to instability [117]. The microencapsulation techniques [118] and some biodegradable polymer-based delivery systems such as liposomes, solid lipid nanoparticles, nanostructured lipid carriers, and emulsions are employed to improve the antioxidants’ bioactivity in cosmetic products [65].

### 3.1. Vitamin C

**Figure 1:**
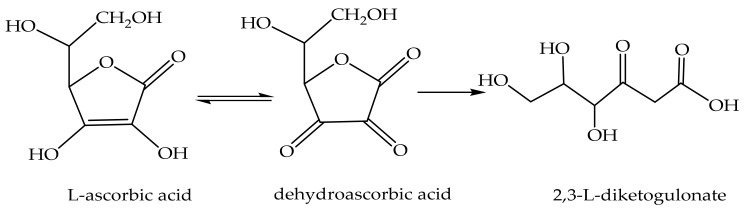
Ascorbic acid oxidation reaction.

Vitamin C, also known as L-ascorbic acid, is a water-soluble vitamin used in skincare and skin lightening products such as antioxidants, skin repair, and connective tissue repair [119,120]. It, especially when exposed to air, oxidizes to dehydroascorbic acid and hydrolyzes to form 2,3-L-diketogulonate (at alkaline pH) [121] (Figure 1).

Therefore, in cosmetic and food fields, some preferred ester derivatives (i.e., retinyl ascorbate, ascorbyl palmitate) encapsulate into microemulsions, polymeric nanoparticles, bilayer vesicles, and solid lipid nanoparticles [122]. Some applications use phosphatidylcholine/lecithin liposomes [123] to enhance the penetration of vitamin C.

### 3.2. Vitamin A

**Figure 2:**
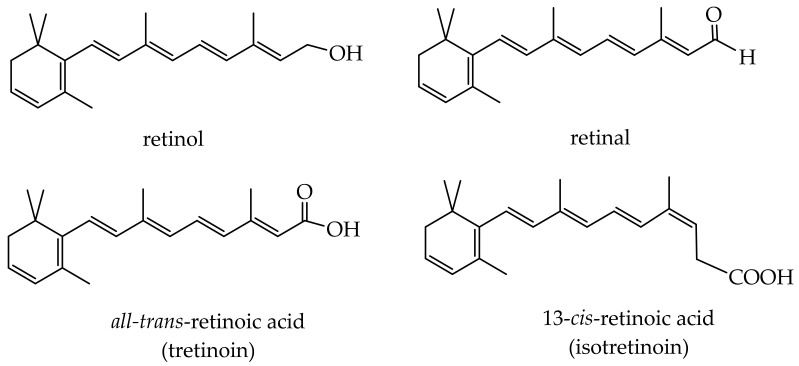
Structures of the most widespread retinoids.

Vitamin A is a fat-soluble vitamin belonging to the group of substances structurally related to the retinol (Figure 2). Vitamin A and retinoids improve wound healing; prevent acne, skin aging, and psoriasis; affect keratinization, keratinocyte proliferation, and epidermal differentiation; and reduce oxidation and inflammation [124]. The retinoids are encapsulated into caprolactone-based nanocapsules and liposomes to protect the bioactive compounds from photodegradation [125,126]. Retinol is encapsulated into silicon particles to prepare antiaging and antiacne formulations [127]. Nanospheres are optimized for the encapsulation of adapalene (third-generation retinoid) to deliver the bioactive to hair follicles [128].

### 3.3. Phenolic Compounds

**Figure 3:**
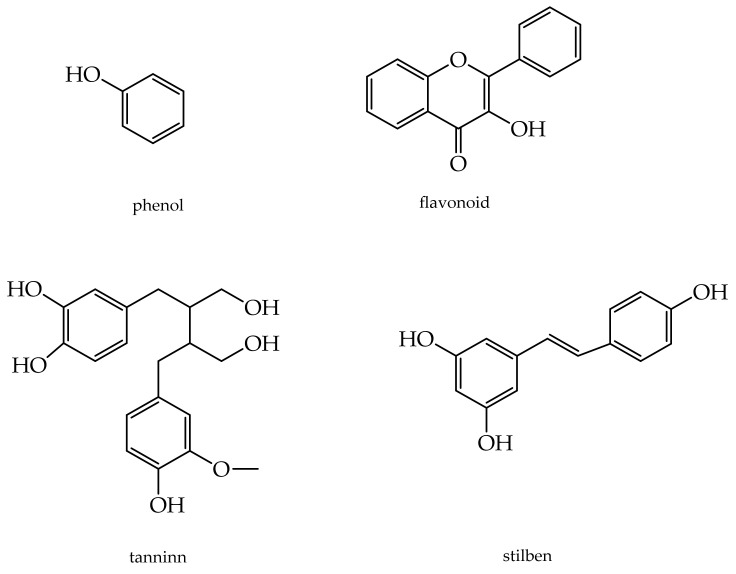
Structures of the most widespread phenols.

Phenolics can be divided into flavonoids (including flavonols, flavones, catechins, flavanones, anthocyanidins, and isoflavones), phenolic acids, stilbenes, coumarins, and tannins (Figure 3).

Phenolic compounds control skin inflammation, wound healing, and barrier homeostasis [129,130]. Phenolics can scavenge metal ions, block pathogenic free radicals, and induce the expression of protective genes against oxidative stress [10]. For example, the isoflavone genistein is used as an anti-wrinkle to protect and hydrate the skin and decrease the oxygen free radical, the expression of MMP-1, and inducible nitrogen oxide synthase [131,132].

Unfortunately, low levels of aqueous solubility, poor gastrointestinal stability, low absorption due to passive diffusion and active efflux in the gastrointestinal tract, and lack of target specificity in the human body limit their application. Examples of nanosystems used to enhance the delivery and bioavailability of polyphenols are nanocapsules, solid lipid nanoparticles, niosomes, and microemulsions. MethoxyPEG-palmitate nanocapsules and chitosan particles are used to load curcumin, the chitosan-tripolyphosphate nanoparticles to encapsulate epigallocatechin-3-gallate [133], and polymeric nanocapsule suspensions are employed in topical formulations directly applied on the skin or as an ingredient in semisolid formulations. The nanocapsules have a pH (slightly acid) similar to the skin pH; they can form a thin film, which causes long-term delivery [134]. The solid lipid nanoparticles are employed to decrease resveratrol photodegradation, improve its cellular uptake, and internalization in keratinocytes [135]. Niosomes improve the antioxidant activity and the skin permeation of ascorbic acid, ellagic acid, curcumin, and resveratrol [136]. Microemulsions, lipid nanoparticles, and liposomes are exploited to enhance the anti-skin-aging properties of the soy’s isoflavones [85]. Some polyphenols used in the cosmetic field have been encapsulated in nanoparticles made by ionic gelation and microspheres produced by spray-drying [137].

### 3.4. Coenzyme Q10

Coenzyme Q10 (CoQ10) is a lipophilic antioxidant synthesized in humans employed in cosmetics formulation as an anti-photo-aging bioactive. It improves fibroblast proliferation and protects lipid membranes and DNA against oxidative damage, acting as a radical scavenger [138]. Liposomes, lipid nanoparticles, and solid nanoparticles are used to improve penetration in the deeper layers of the skin [139]. Farbound et al. incorporated CoQ10 into SLNs and then into a semisolid emulsion in a cosmetic formulation to improve skin elasticity and hydration [140]. El-Leithy et al. formulated a nanoemulsion containing CoQ10 as an anti-wrinkle cosmetic product [141]. Pegoraro et al. formulated a topical cosmetic product to decrease the effects of UVB radiation, encapsulating CoQ10 and vitamin E in caprolactone nanocapsules and incorporating them into hydrogels [142].

### 3.5. Terpenoids (Also Called Isoprenoids)

**Figure 4:**
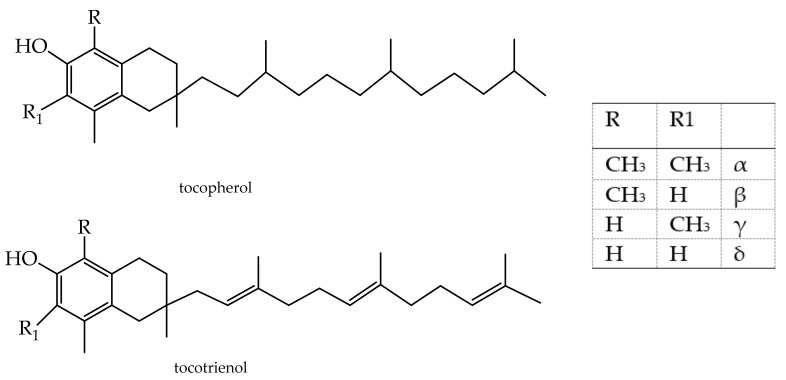
Tocopherol and tocotrienol.

The terpenoid subclasses are tocotrienols and tocopherols (Figure 4). They are derived from five-carbon isoprene units, which differ in carbon skeletons and functional groups. Most are multicyclic structures.

The terpenes react with free radicals by partitioning themselves into fatty membranes. The tocopherols are used in cosmetic formulations as skin-conditioning agents or antioxidants [143] to control wound healing, skin inflammation, and barrier homeostasis [129,130]. Tocopherol is a skin irritant and light-sensitive liquid. Therefore, it is placed into nanocarriers to produce a cosmetically appealing formulation. The best performances are obtained using nanostructured lipid carriers and retinol-encapsulated chitosan nanoparticles (i.e., zein-chitosan and succinic-chitosan nanoparticles). The antioxidant activity of the encapsulated retinol is significantly greater than pure retinol [144,145,146,147]. The tocotrienols are oral care agents, light stabilizers, and skin-conditioning agents; a-tocopherol acetate is the most widespread vitamin E used in commercial sunscreen and skincare products. It is used as a preservative. Both α-tocopheryl acetate and a-tocopherol are used to make supplements, since they are generally recognized as safe (GRAS) food ingredients. Their daily intake varies from 0.15 to 2 mg/kg for DL-α-tocopherol and D-α-tocopherol [143]. Fullerene (carbon-based vehicle) nanocapsules with ascorbic acid and vitamin E have been employed to improve skin protective activity against premature aging [148].

### 3.6. Carotenoids (Alpha-Carotene, Beta-Carotene, Lycopene, Phytoene, Phytofluene)

**Figure 5:**
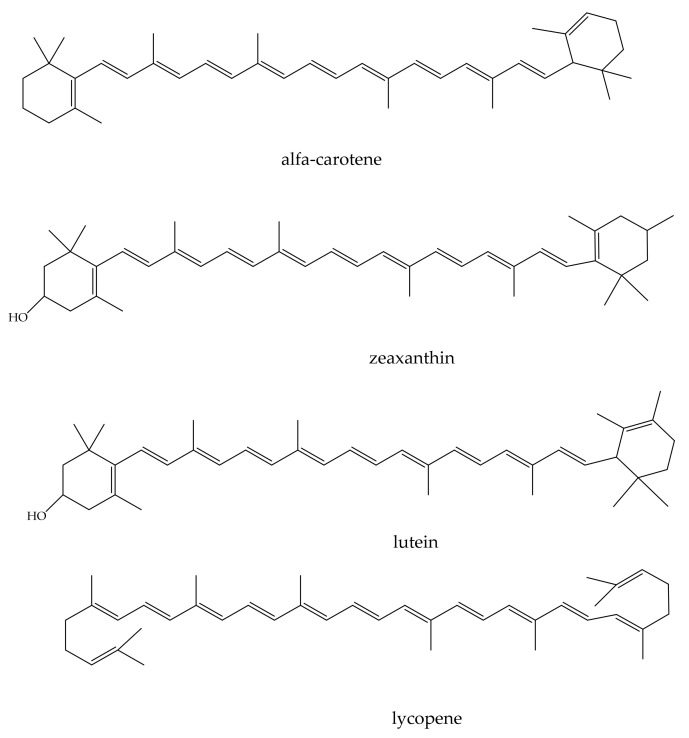
Structures of the most widespread carotenoids.

The carotenoids (i.e., lutein, zeaxanthin, α-carotene, and lycopene) (Figure 5) are natural colorants that can reduce oxygen singlets, stabilize other antioxidants, and block free radicals.

The carotenoids protect against cancers (i.e., uterine, prostate, breast, colorectal, and lung), cardiovascular illnesses, and eye (i.e., age-related macular degeneration and cataracts) and skin disorders [149,150,151,152,153]. The retinoic acid and derivatives modulate, at the skin level, the expression of genes implicated in cellular differentiation and proliferation. The carotenoids act as photoprotective agents against UV radiation. For example, the oral use of the astaxanthin increases skin condition and decreases skin hyper-pigmentation, improving some antioxidant enzymes (i.e., superoxide dismutase, catalases enzyme) activity, and suppressing tyrosinase action [153,154]. The β-carotene protects from sunburn diseases (inhibiting metalloproteinase activation and increasing 5-α-hydroperoxide synthesis) and prevents wrinkle formation and skin flaccidity [155,156]. Carotenoid intake improves skin yellowness and pigmentation [157]. Zeaxanthin enhances the antioxidative defense system and the skin’s hydration and elasticity [158,159,160]. Phytoene and phytofluene have skin whitening effects [152]. Astaxanthin (a xanthophyll carotenoid) was loaded in poly(D,L-lactic-co-glycolic acid) (PLGA) nanoparticles by using the emulsion solvent evaporation technique to improve its antioxidant and anti-wrinkle effects [161].

### 3.7. Organosulphur Compounds

**Figure 6:**
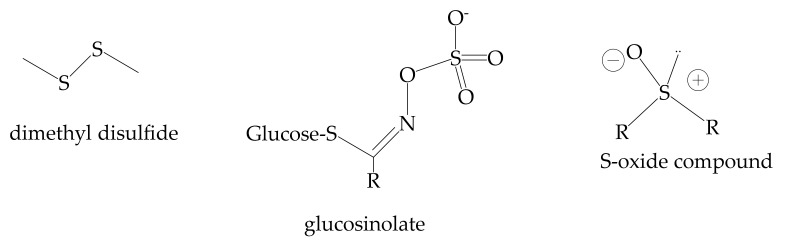
Structures of the most widespread organosulphur compounds.

The organosulphur compounds are organic substances classified according to the functional groups containing sulfur atoms in thiols, sulfides, sulfoxides, sulfones, thiosulfinates, sulfimides, sulfoximides, sulfonediimines, glucosinolates, thioketones, thioaldehydes, S-oxides and S,S-dioxides of thiocarbonyl compounds, thiocarboxylic acids, thioamides, sulfonic, sulfinic, sulfenic acids, and related compounds, sulfuranes, and persulfuranes (Figure 6) [162].

They have anti-atherosclerosis, antifungal, antimicrobial, immunostimulating, and antithrombotic activities [162]. In cosmetic formulations, they are used as antioxidants and skin-conditioning agents. Some organo-sulfur compounds (i.e., 1-propylmercaptan, 2,5-dimethylthiophene, dimethyl disulfide, diallyl disulfide, and propyl disulfide) decrease tyrosinase activity and melanin development, reducing the reactive oxygen species and improving the glutathion/glutathione disulfide ratio in B16 cells [163]. The administration of broccoli extract (rich in glucosinolates) decreases skin lesions and improves keratin production. Sulforaphane decreases the risk of skin lesions caused by UV radiation [164]. Allicin cures premature aging, inhibiting a leukocyte elastase [165]. The pegylated liposomes and nanoparticles (e.g., diallytrisulfide-polybutyl cyanoacrylate-nanoparticles) have been employed to deliver the organosulphur compounds [166]. An anti-dandruff shampoo was formulated using SLNs of garlic as an antifungal agent [136].

### 3.8. Methylxanthines (Theophylline, Caffeine, and Theobromine)

**Figure 7:**
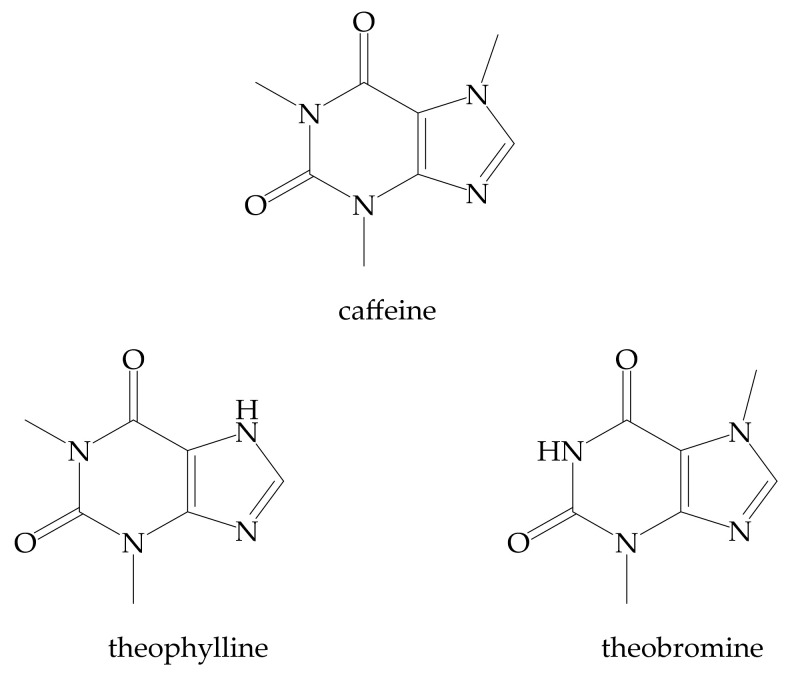
Structures of the most widespread methylxanthines.

The methylxanthines are alkaloids (purine base) used in cosmetic formulations as scavengers of reactive oxygen species generated by UV exposure, antiaging, and anti-cellulitis agents (Figure 7). Caffeine is an antiaging, photoprotective, antioxidant, anticellulite, and antiacne compound [167]. Unfortunately, it can precipitate and form clumps when used in topical applications. [168]. Solid lipid nanoparticles have been designed to solve these problems [169].

## 4. Nanodelivery System Toxicity

The nanodelivery systems’ different particle sizes, surface groups, zeta potentials, and aggregation states can determine different bioavailability and toxic reactions than conventional ones [170]. The smaller size of nanomaterials allows them easier access into cells, tissues, and organs, decreasing the influence of intestinal clearance mechanisms and protracting their stay in the gastrointestinal tract [171]. Therefore, the tolerable upper intake levels (UL) and recommended daily allowance (RDA) of nutrients need reevaluation [172]. Furthermore, the nanoparticles interact with various immune system components, breaking up immunostimulation or immunosuppression, promoting inflammation and autoimmune disorders, or increasing the host’s susceptibility to infections. They can interact with the innate and adaptive immune systems [173]. The innate immune system is our first line of defense against invading organisms and can immediately respond to any stress. It consists of cells (e.g., physical epithelial barriers, phagocytic leukocytes, dendritic cells, natural killer cells) and proteins (e.g., circulating plasma proteins) that are always present and ready to mobilize and fight microbes at the site of infection. The innate immune system is nonspecific and has no memory. The adaptive immune system acts as a second line of defense and can respond efficiently to re-exposure to the same pathogen. The components of the adaptive immune system are generally silent; however, when activated, these components “adapt” to the presence of infectious agents by activating, proliferating, and creating potent mechanisms for neutralizing or eliminating the microbes. There are two adaptive immune responses: humoral immunity, mediated by antibodies produced by B lymphocytes, and cell-mediated immunity, mediated by T lymphocytes. The nanoparticles can elicit an immune response by directly immunostimulating antigen-presenting cells or delivering antigen to specific cellular compartments. Their compatibility with the immune system depends on size, surface charge, hydrophobicity/hydrophilicity, and steric effects of the particle coating. Predicting nanoparticles’ innate responses (in vitro or in vivo) is still challenging [10]. Some nanomaterials induce a (pro)inflammatory response and are taken up by phagocytic cells, whereas others seem to reduce these activities, reducing the ability of these immune cells to fight (e.g., bacteria).

Moreover, nanomaterials can affect the adaptive immune response, disrupting the Th1/Th2 balance, influencing cytokine production in peripheral blood mononuclear cells, and overproducing TNF-α (tumor necrosis factor) and INFγ interferon, decreasing levels of IL-10 (interleukin) and IL-2. It is possible to show an overt immune response when nanoparticles are designed with poly(ethylene glycol) (PEG) or other types of polymers to provide a hydrophilic environment [174,175]. The silver, titanium dioxide, zinc, and zinc oxide nanoparticles are highly toxic, since their high surface area increases the contact with biomolecules and triggers adverse responses. Cationic charge determines a high affinity towards the negatively charged plasma membrane, which determines retention of one Cl^−^ ion and one water molecule per proton and consequent lysosomal swelling and rupture [176]. The toxicity of nanoceuticals was studied mainly in animal experiments. More human and clinical trials should be carried out to know the potential positive and negative effects of nanoceutics and/or nanocosmetics on human health [177].

## 5. Conclusions

The use of nanotechnologies represents an excellent opportunity to improve the performance of nutricosmeceutic products. Nanodelivery systems improve bioactive compounds’ bioavailability and water solubility, guarantee their release at the site of action, mask their unpleasant taste, and prolong their expiration date. Unfortunately, only a few studies on animals and very limited on humans have investigated their potential toxic effects. More human and clinical trials should be carried out to protect consumer health.
